# Supplementary material for: Potent neutralization by monoclonal human IgM against SARS‐CoV‐2 is impaired by class switch
Source: EMBO Rep. 2022 May 12;23(7):e53956. doi: 10.15252/embr.202153956 (PMC9253785; doi:10.15252/embr.202153956)
Supplement: Supplementary file 1 — Appendix [file EMBR-23-0-s005.pdf]

Appendix for 'Potent neutralization by monoclonal human IgM against SARS-CoV-2 is impaired by class switch'.

Page number	Appendix Table	Legend
Page 2	Appendix Table S1	Demographic features of donors for serum testing and spike-specific B cell sorting.
Page 3	Appendix Table S2	Characteristics of donors from whose B cells monoclonal antibodies were isolated. Sex: "m"= male. Disease course designations are as follows: Mild: loss of smell/taste, mild fever, elevated temperature. Intermediate: fever, cough, chest pain, mild dyspnea, no need for hospitalization.
Page 4	Appendix Table S3	Heavy and light constant region, V-D-J usage, amino acid changes and CDR3 sequences of cloned spike-binding antibodies.
Page 5	Appendix Table S4	Marker panel used for MACACS phenotyping.
Page 6	Appendix Table S5	SPR antibody binding affinities to recombinant spike protein. All measurements were made with antibodies expressed as IgG1. Full extracellular domain of spike protein was used as antigen when this was stably bound. When binding to full spike ECD was not stable, recombinant subunits based on the ELISA results (Fig 5A) were used instead (last column).
Page 7	Appendix Table S6	Primer sequences used in amplifying immunoglobulin genes from cDNA.
Page 8	Appendix Table S7	Primer sequences used in switching class from IgM to IgG1, from IgA to IgM and from IgG to IgM.

Appendix Table S1

	Healthy donor	Convalescent	Post-vaccination
Total number	86	34	14
Mean age	47 years (21-83)	43 years (22-75)	38 years (22-60)
Gender	43 F (48%)	8 F (24%)	6 F (43%)
Mean time from infection/vaccination		39 days (12-71)	24 days (13-45)

Appendix Table S2

ID	Age	Sex	Date of donation	Onset symptoms	Disease course	B cells obtained for sorting	Spike-capturing B cells sorted and expanded	Spike-specific antibodies produced
F2978	41	m	30.Mar.2020	23.Feb.20	mild	483’555	954	198
F3005	40	m	23.Apr.20	16.Mar.20	intermediate	388’978	1094	41
F3007	59	m	*24.Apr.20*	13.Mar.20	mild	127’859	190	30
F3010	46	m	30.Apr.20	10.Mar.20	intermediate	259’627	273	26
F3015	26	m	06.May.20	17.Mar.20	mild	181’083	755	170

Appendix Table S3

			Heavy chain					Light chain				
**Donor ID**	**Antibody**	**Heavy constant**	**V**	**D**	**J**	**aa changes**	**HCDR3**	**Light constant**	**V**	**J**	**aa changes**	**LCDR3**
F3015	1B17	alpha 1	1-69*01	5-18*01	4*02	7	ATWSDVDTDMFFDY	kappa	3-11*01	2*01	3	QQRSNWPPYT
F2978	1G22	alpha 1	1-69*01	3-22*01	4*02	4	AITSDYDSSGYYSDY	kappa	3-11*01	2*01	0	HQRSNWPPMYT
F2978	2B15	alpha 1	4-59*01	N/A	4*02	0	ARGFDY	kappa	3-20*01	1*01	4	QQYGSSPPWT
F2978	2C20	alpha 1	1-2*02	3-22*01	3*02	1	ARVSYFYDNSGYPGAFDI	lambda2	2-14*01	3*02	4	SSYTSSSTWV
F2978	2J12	alpha 1	3-30-3*01	1-26*01	4*02	3	ARDRSGSYTYFDY	kappa	3-11*01	4*01	0	QQRSNWLT
F3015	3M13	alpha 1	3-30*18	1-26*01	3*02	5	AKPRSGNYHDAFDI	kappa	3-20*01	2*01	1	QQYGSSYT
F2978	1G4	gamma 1	5-51*01	3-9*01	6*02	0	ARLNFDYLSGYYYYYGMDV	kappa	3-20*01	4*01	0	QQYGSSRGT
F2978	1I12	gamma 1	3-30*04	3-16*01	6*02	5	AKDQVEVEPILMMFGGLTTASVGDV	kappa	3-15*01	4*01	1	QQYNNWPPLT
F2978	1M18	gamma 1	1-2*02	3-3*01	4*02	1	ARDERSGDFWTAYYYHFDY	kappa	1-12*01	5*01	0	QQANSFPLT
F2978	2O17	gamma 1	3-30*03	1-26*01	3*02	2	ARPYSGSYWSAFDI	lambda	1-51*01	2*01	12	AAWDTSLSGVV
F3005	2N5	gamma 1	3-30*18	3-22*01	4*02	3	AKEGRGPYYYDSSGYFDY	kappa	1-33*01	4*01	0	QQYDNLPT
F3005	3H9	gamma 1	2-5*01	5-12*01	6*02	4	VHGYSWYYYGMDV	lambda	2-14*03	2*01	7	SSYTASSTLGV
F3007	1K11	gamma 1	4-59*01	2-8*01	6*02	11	ARVTRYCTKGVCQTDYFDGMDV	lambda1	1-40*01	1*01	8	QSYDGSLGGV
F3010	1B11	gamma 3	3-30*03	1-26*01	4*02	5	AQADSGGYYSSYY	kappa	6-21*01	4*01	1	HQSSSLPHT
F3010	1I7	gamma 1	1-18*04	2-2*02	6*02	2	ARDGPFTVVVPTAIRDQVDYYYYGMDV	kappa	2-30*01	2*01	4	MQGTHWPPYT
F3015	1J12	gamma 1	3-30*18	2-2*01	5*02	2	AKAKDIVVLPALNWFDP	lambda1	2-14*01	1*01	0	SSYTSSSTPFV
F3015	2K21	gamma 1	4-4*07	N/A	5*02	8	AMGFDP	kappa	3-20*01	1*01	2	QQYGSSPWT
F2978	2J17	mu	1-2*02	4-11*01	4*02	3	ARSNHYTNPVVRLDY	kappa	1-33*01	4*01	1	QQYDNLPPKLT
F3005	2E14	mu	3-15*01	2-15*01	4*02	2	IPRPSWYCSGDNCPSYGSAAEHDY	lambda2	3-1*01	3*02	4	QAWDSSTEA
F3005	3N8	mu	3-23*01	4-23*01	2*01	9	ARGGGPYWYFDL	lambda	2-23*01	2*01	2	CSYAGSTTHVV

Appendix Table S4

Manufacturer	Catalogue	Marker	Clone	Fluorophore
BD Pharmingen	561295	CD19	H1B19	PERCPCy5.5
BD Phosflow	563347	CD20	H1	BV510
BD Horizon	563163	CD21	B-ly4	BV711
BD Horizon	562513	CD27	M-T271	BV421
eBioscience	47-0389-42	CD38	HIT2	APC-eFluor780
Biolegend	310932	CD69	FN50	BV785
Biolegend	564606	CD138	MI15	FITC
Miltenyi	130-113-998	IgA	IS11-8E10	APC
BD Pharmingen	561314	IgD	IA6-2	PE-Cy7
BD Pharmingen	561296	IgG	G18-145	Alexa700
BD Horizon	562977	IgM	G20-127	BV605
Molecular Probes	C34557	donor cell label		cell trace violet
		spike protein		mCherry

Appendix Table S5

Ab	original class	tested class	epitope (ELISA)	Kd (M)	antigen
2E14	IgM	IgG	NTD	3.56E-09	Spike
2J17	IgM	IgG	RBD	2.89E-09	Spike
3N8	IgM	IgG	RBD	4.98E-09	Spike
1I12	IgG	IgG	NTD	1.08E-08	Spike
1M18	IgG	IgG	RBD	2.82E-13	Spike
1B11	IgG	IgG	S2	7.54E-10	Spike
2N5	IgG	IgG	RBD	1.37E-10	RBD
3h9	IgG	IgG	S2	7.63E-11	S2
1I7	IgG	IgG	S2	3.54E-09	S2
1J12	IgG	IgG	S2	7.48E-12	S2
2K21	IgG	IgG	S2	9.92E-11	S2

Appendix Table S6

Heavy chain forward

V gene	forward primer name	forward primer sequence
3-30	H_3-30_5UTR_for	TAGAAGTCGGCGGTGTTTCC
5-51	H_5-51_5UTR_for	CTGGGATCTCAGGGCTTCAT
4-59	H_4-59_5UTR_for	ACCTCCTGTGCAAGAACATGA
1-69	H_1-69_5UTR_for	TCCTCCTCTAAAGAAGCCCCT
3-15	H_3-15_5UTR_for	TCCCAAGTGTTTTCATTCAGTG
1-18	H1-18_for_Age_or_Bsp	caaagcaTCCGGAACCGGTctcaccATGGACTGGACCTGGAGC AT
1-2	H_1-2_5UTR_for	AATCCCCTGAGAGCTCCGTT
4-4	H_4-4_5UTR_for	GGACCTCCTGCACAAGAACA
2-5	H_2-5_5UTR_for	AGTGACTCCTGTGCCCCA
3-23	F3005-3N8_mu_3-23_for	tcaaagcaaccggtgatatcaCTGGGATTTTCAGGTGTTTTCA

Light Chain forward

V gene	forward primer	forward primer sequence
2-30	kappaV2-30_for_BspEI	ttcaaagcaaTCCGGAcctcagttcaccttctcacaATG
1-33	K_1-33_5UTR_for	GTGGTATCAACGCAGAGTACG
1-40	L_1-40_5UTR_for	AGAGGCAGCACTCAGGACAA
1-51	L_1-51_5UTR_for	TCTTCATCATGACCTGCTCCC
2-14	L_2-14_5UTR_for	CAGCGCTCTCAGGACATCTC
3-15	K_3-15_5UTR_for	GCTCAGTTAGGACCCAGACG
3-20	K_3-20_5UTR_for	GCTGCTCAGTTAGGACCCAG
3-1	F3005-2E14_lambda_3-1	CTCAGGAAGCAGCATCGGAG
1-5	K_1-5_5UTR_for	GGAGTCAGACCCAGTCAGGA
6-21	kappaV6-21_for_BspEI	ttcaaagcaaTCCGGAgccaccATGTTGCCATCACAACTCATTGG GTTTCTGCTGC
3-11	K_3-11_5UTR_for	TAGGACCCAGAGGGAACCAT
1-12	K_1-12_5UTR_for	CAGTCAGGACACAGCATGGA
2-23	F3005-3N8_lambda_2-23_for	CACAAGAGGCAGCGCTCT
2-28	K_2-28_5UTR_for	CTCACAATGAGGCTCCCTGCT

Heavy chain reverse

Reverse primer name	Reverse primer sequence
mu_3pUTR_rev_MCS2	tcatgtctggccagctagctATGCAACATCTCACCCCGTT
gamma_3pUTR_rev_MCS2	tcatgtctggccagctagctTGGGTGCTTTATTTCCATGCTG
alpha_3pUTR_rev_MCS2	tcatgtctggccagctagctAGCATGGAGTTTATTCAGGGGT

Light chain reverse

reverse primer name	reverse primer sequence
kappa_3pUTR_rev_MCS1	tcctaggcgtacgggatccGGGTGAGGTGAAAGATGAGCT
lambda_3pSTOP_rev_MCS1	tcctaggcgtacgggatccCTATGAACATTCTGTAGGGGCCACTGTCTT

Appendix Table 7

Class switch primers

Primer name	Primer sequence
IGHJ4_Gcon_fus_rev	gaccgatgggcccttggtggaggcTGAGGAGACGGTGACCAGG
J3_02_rev_fus_to_mu	gaaaagggttggggcggatgcactcccTGAAGAGACGGTGACCATTGT
J6_02_rev_fus_to_mu	gaaaagggttggggcggatgcactcccTGAGGAGACGGTGACC
pVITRO_MCS2_for_out	tgagtcacccacacaaagga
pVITRO_MCS2_for_in	ctaattcaaagcaaccggtgat
pVITRO_MCS2_rev_out	aaaacctcccacacctccc
pVITRO_MCS2_rev_in	tcactgcattctagttgtggt
gamma1_constant_for	gcctccACCAAGGGCCCATCGGTC
