# Supplementary material for: Potent neutralization by monoclonal human IgM against SARS‐CoV‐2 is impaired by class switch
Source: EMBO Rep. 2022 May 12;23(7):e53956. doi: 10.15252/embr.202153956 (PMC9253785; doi:10.15252/embr.202153956)
Supplement: Supplementary file 2 — Expanded View Figures PDF [file EMBR-23-0-s004.pdf]

Expanded View Figures

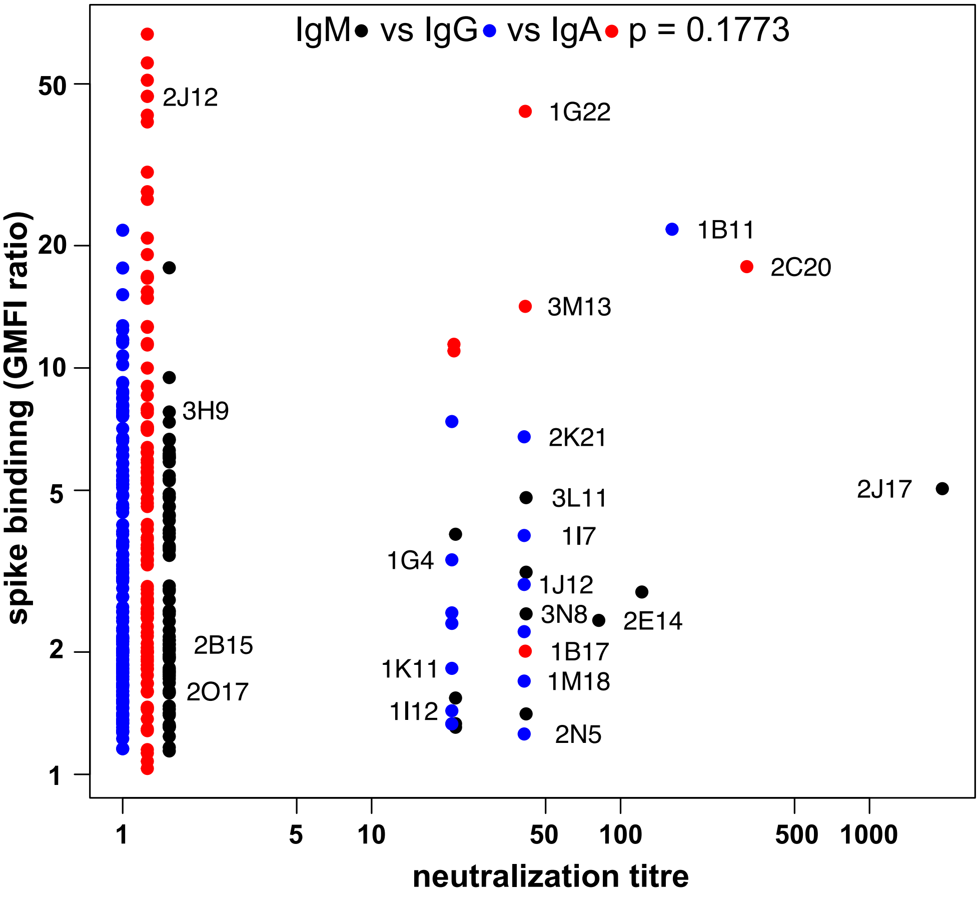

Figure EV1. Spike binding and virus neutralization by single well supernatants.

Supernatants shown in (B) that exhibited a ratio of fluorescent signal on spike-expressing cells to signal on non-expressing cells > 1.2 (n = 326: 79 IgM, 141 IgG 106 IgA) were assayed for ability to neutralize SARS-CoV-2 pseudotyped VSV. Supernatants were serially diluted from 20–1,960 fold, and mixed with 100 pfu of VSV*ΔG-$S_{\Delta 21}$, which was then used to infect Vero cells. Assay was performed once for each supernatant in quadruplicate, and the titer defined as the maximum dilution at which virus proliferation was completely inhibited. For each supernatant tested, the GMFI as shown in (B) is shown on the vertical axis, and the neutralization titer is shown on the horizontal axis, slightly staggered by class, in order to minimize overlap of points. IgM, IgG, and IgA are plotted with black, blue, and red points respectively. *P* values at the top of the plot were calculated by chi-square test of the null hypothesis that the likelihood that supernatants show a titer of 20 (the lowest dilution tested) or greater is equal among the three antibody classes.

Figure EV2. Spike and epitope binding by monoclonal antibodies.

A Spike recognition by monoclonal antibodies assessed by flow cytometry. Each contour plot shows mCherry on the horizontal axis, so that spike-mCherry expressing cells are on the right of the plot, and TE 0 cells on the left. Vertical axes show signal of secondary antibodies used to detect IgM, IgA, or IgG.

B Heatmap of antibody binding to spike protein subdomains (complete spike extracellular domain "spike", S1, S2, RBD and NTD) in ELISA. Color gradient correspond to the OD of each sample. The top three rows show binding of native IgM. The lower three rows show binding of the same antibodies, expressed as IgG1.

▶

 © 2022 The Authors

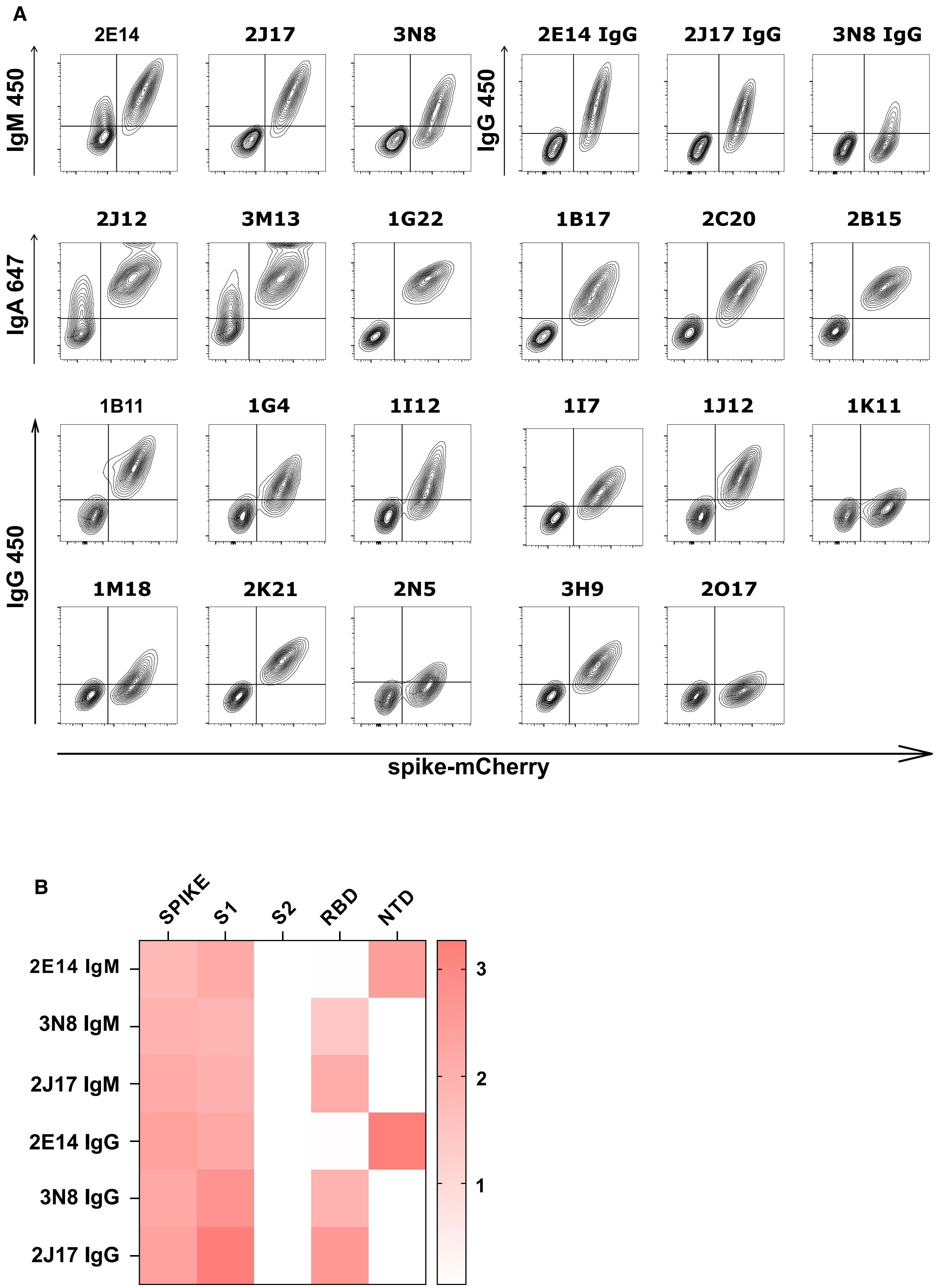
A
2E14
2J17
3N8
2E14 IgG
2J17 IgG
3N8 IgG
IgM 450
IgG 450
2J12
3M13
1G22
1B17
2C20
2B15
IgA 647
1B11
1G4
1I12
1I7
1J12
1K11
1M18
2K21
2N5
3H9
2O17
IgG 450
spike-mCherry
B
SPIKE
S1
S2
RBD
NTD
2E14 IgM
3N8 IgM
2J17 IgM
2E14 IgG
3N8 IgG
2J17 IgG
3
2
1

Figure EV2.

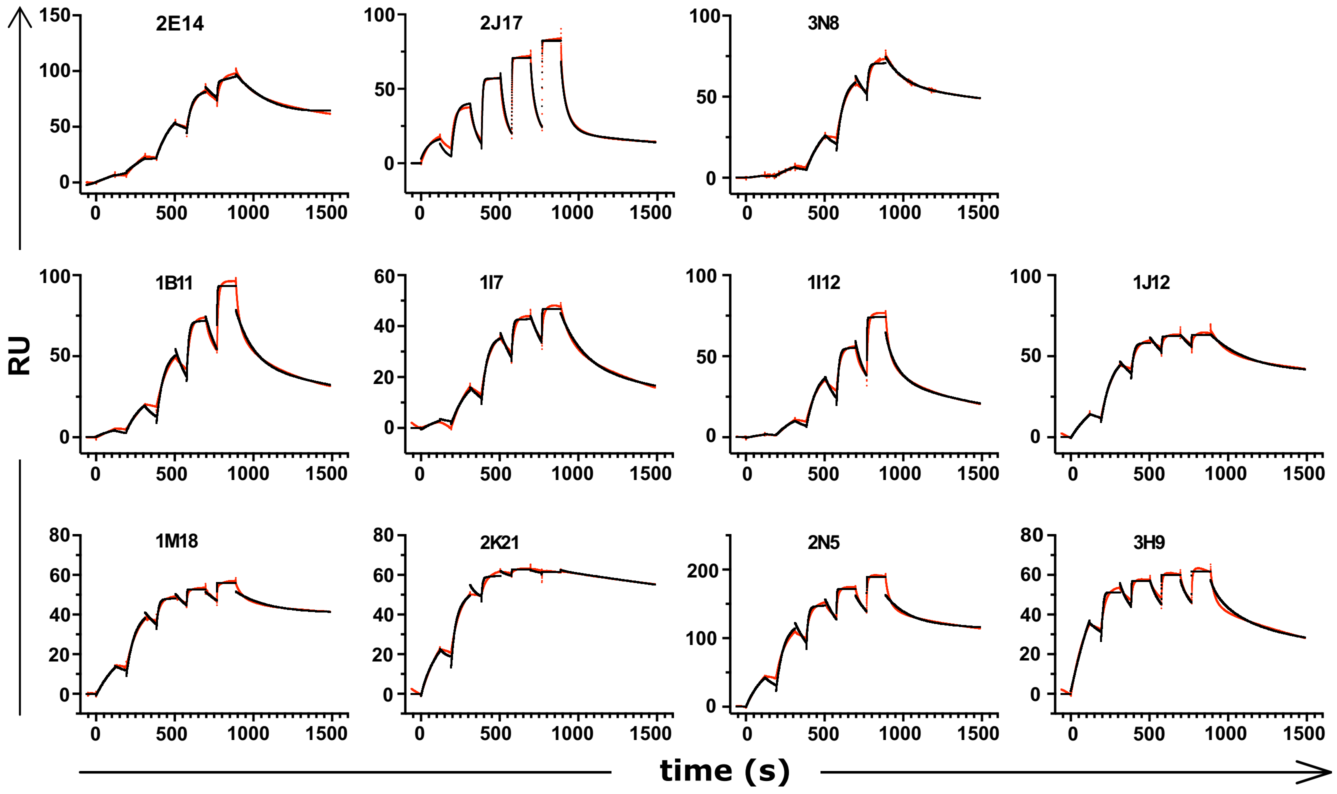

Figure EV3. Surface plasmon resonance results.

Sensograms derived from surface plasmon resonance affinity measurement of donor-derived spike-binding IgG1 (n = 8) and donor-derived IgM (n = 3) expressed as IgG1.

© 2022 The Authors

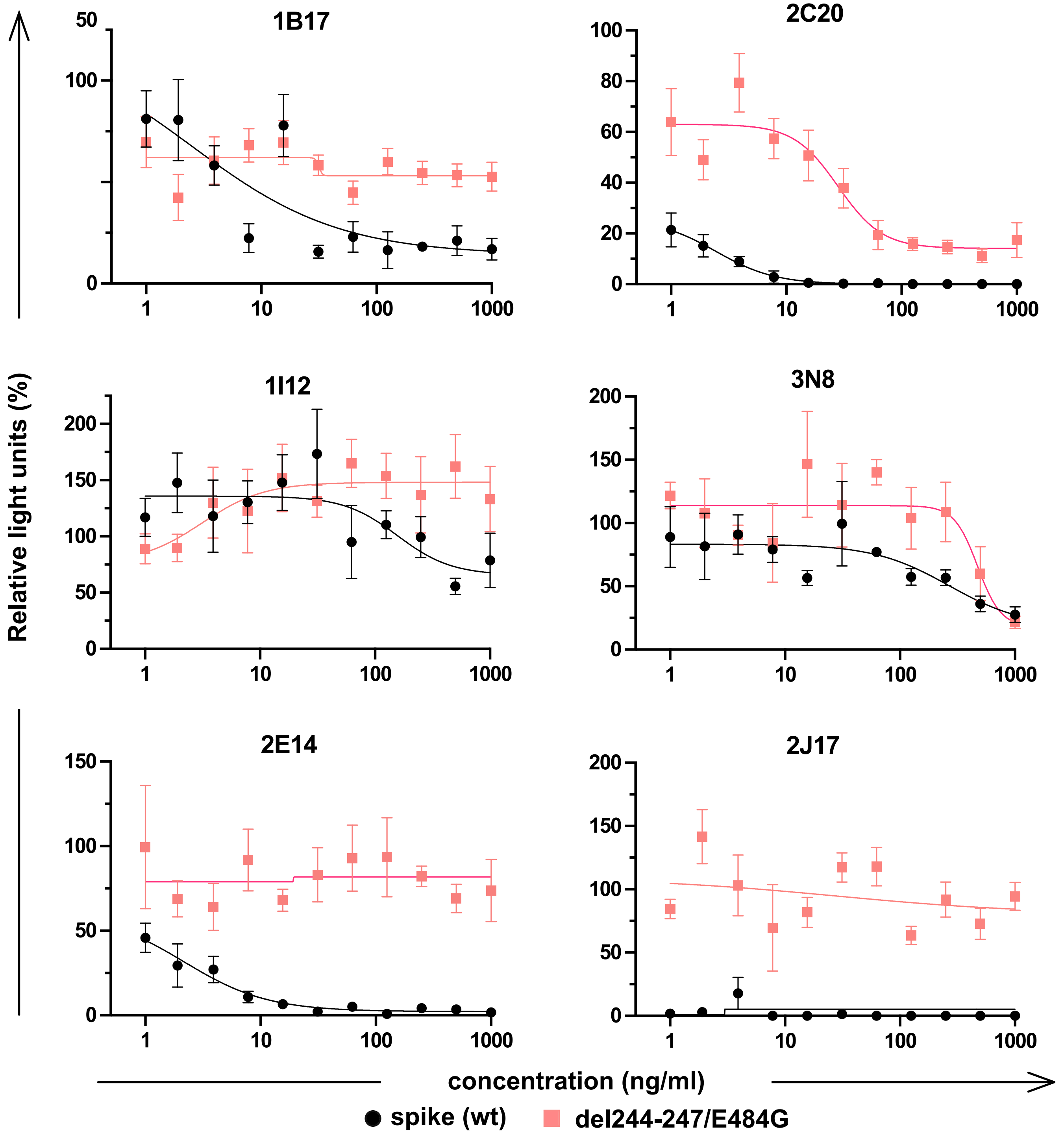

Figure EV4. Comparison of neutralization of VSV pseudotyped with wild-type or mutant spike protein by six monocloal antibodies.

Neutralization curves of 3 IgM, 1 IgG, and 2 IgA using VSV*ΔG(FLuc) pseudotyped with wild-type (wt) spike protein or with spike variant harboring the deletion of amino acids 244-247, located in the NTD, and the substitution E484G, located in the RBD, from a SARS-CoV-2 isolate from an immunosuppressed COVID-19 patient (del244-247/E484G). The horizontal axis shows the serial dilution of the antibodies. The vertical axis shows, for each well exposed to antibody, the percentage of the relative light unit of the control wells without antibody. Each point is the mean of 4 values pooled from three independent experiments. Error bars indicate standard error.
